# Association of Intrapartum Cardiotocography Findings with Umbilical Arterial Blood Gas Parameters and Neonatal Outcomes in Non-Reassuring Fetal Status

**DOI:** 10.3390/jcm15145464

**Published:** 2026-07-13

**Authors:** Bilge Çetinkaya Demir, Aylin Orhaner

**Affiliations:** 1 Department of Perinatology, Faculty of Medicine, Bursa Uludag University, 16059 Bursa, Turkey; 2 Department of Obstetrics and Gynecology, Medicana Hospital, 16059 Bursa, Turkey

**Keywords:** intrapartum cardiotocography, fetal well-being, non-reassuring fetal status

## Abstract

**Objectives**: To investigate the relationship between intrapartum cardiotocography (CTG) findings and neonatal outcomes by evaluating the association between CTG tracings, umbilical cord blood gas parameters, and APGAR scores, and to assess the diagnostic performance of CTG in identifying fetuses at risk of neonatal acidemia. **Methods**: This prospective study included women who delivered at a tertiary teaching hospital between January 2017 and January 2018. Of 1100 women initially screened, 596 met the inclusion criteria and were analyzed. Among them, 162 underwent operative delivery for non-reassuring fetal status (NRFS), while 434 served as controls. Demographic, obstetric, and neonatal characteristics were compared. Multivariable logistic regression was performed to explore factors associated with umbilical arterial pH < 7.20. **Results**: Mean umbilical arterial pH was significantly lower in the NRFS group than in controls (7.30 ± 0.08 vs. 7.32 ± 0.07, *p* < 0.05). Umbilical arterial pH differed significantly across NICHD fetal heart rate categories, with the highest values observed in Category I tracings. Twenty-eight neonates (4.7%) had an umbilical arterial pH < 7.20. CTG-based identification of NRFS predicted umbilical arterial pH < 7.20 with a sensitivity of 42.9%, specificity of 73.6%, positive predictive value of 7.4%, and negative predictive value of 96.3%. In multivariable analysis, pregestational diabetes mellitus and preeclampsia were independently associated with umbilical arterial pH < 7.20. **Conclusions**: Although CTG remains an essential tool for intrapartum fetal surveillance, its low positive predictive value indicates that many fetuses classified as having NRFS do not have biochemical evidence of acidemia. CTG findings should therefore be interpreted together with the overall clinical context, including maternal risk factors such as pregestational diabetes mellitus and preeclampsia, rather than being used in isolation to guide intrapartum management.

## 1. Introduction

Assessment of fetal well-being is a fundamental component of modern obstetric care. Advances in perinatal medicine have enabled the fetus to be regarded as a patient, emphasizing the importance of timely identification of fetal compromise and appropriate intervention when necessary [1]. Various methods are currently used to evaluate fetal status, including cardiotocography (CTG), biophysical profile assessment, Doppler ultrasonography, umbilical cord blood gas analysis, and neonatal APGAR scoring [2,3].

Among these methods, CTG remains the most widely used tool for intrapartum fetal surveillance. Abnormal CTG findings frequently prompt operative delivery because they are considered indicators of fetal hypoxia and potential acidemia. However, despite its widespread use, the diagnostic accuracy of CTG remains controversial [1]. Randomized studies have demonstrated that continuous CTG monitoring is associated with increased operative delivery rates, particularly cesarean section, without a corresponding improvement in long-term neonatal outcomes [2,4]. Furthermore, considerable interobserver variability in CTG interpretation has been reported, raising concerns regarding its reliability in predicting adverse neonatal outcomes [4,5]. Recent advances in artificial intelligence and machine learning (ML) have shown promising potential to improve the prediction of fetal hypoxia beyond conventional visual interpretation of cardiotocography (CTG). However, current evidence indicates that further validation using diverse datasets, standardized benchmarks, and improved algorithms is required before these approaches can be routinely implemented in clinical practice [6]. Among these models, DeepCTG 1.0 demonstrated moderate predictive performance (AUC 0.74) while reducing the false-positive rate compared with expert clinicians, highlighting the potential of automated CTG interpretation [7]. Nevertheless, umbilical cord blood gas analysis remains the reference standard for objectively assessing neonatal acid-base status and evaluating the diagnostic performance of CTG.

Umbilical cord blood gas analysis is considered the gold standard for the objective assessment of fetal acid–base status at birth. Umbilical arterial pH and lactate measurements provide valuable information regarding intrapartum hypoxia and neonatal well-being and have been shown to correlate with short-term neonatal morbidity [8,9,10]. Low umbilical arterial pH values are associated with an increased risk of neonatal seizures, respiratory support requirements, admission to the neonatal intensive care unit, and perinatal mortality [9,10]. Therefore, cord blood gas analysis offers an objective measure against which the clinical performance of CTG can be evaluated.

Despite ongoing advances in computerized CTG interpretation, evaluating the relationship between CTG-based diagnosis of non-reassuring fetal status and objective neonatal acid-base status remains clinically important. The present study aimed to investigate the association between intrapartum CTG findings, umbilical arterial blood gas parameters, and neonatal outcomes by comparing women with normal CTG tracings and those who underwent operative delivery for suspected NRFS. We also evaluated the diagnostic performance of CTG in identifying fetuses at risk of acidemia during labor. We hypothesized that pregnancies undergoing operative delivery for suspected NRFS would have lower umbilical arterial pH values and less favorable neonatal outcomes than those with normal CTG tracings, and that CTG alone would have limited diagnostic accuracy for predicting fetal acidemia.

## 2. Materials and Methods

### 2.1. Study Design and Population

This prospective observational study was conducted at the Department of Obstetrics and Gynecology, Bursa Uludağ University Faculty of Medicine, between January 2017 and January 2018. Ethical approval was obtained from the Bursa Uludağ University Faculty of Medicine’s Clinical Research Ethics Committee (Approval No.: 2018-17/9).

During the study period, 1100 women delivered at our institution. Of these, 596 met the study eligibility criteria and were included in the final analysis. Maternal demographic and obstetric characteristics were prospectively recorded using patient medical records and the hospital electronic database. The collected variables included maternal age; body mass index (BMI); gravidity; parity; number of previous abortions; gestational age at delivery; neonatal birth weight; umbilical arterial pH; 1, 5, and 10 min APGAR scores; presence of meconium-stained amniotic fluid; mode of delivery; indication for cesarean delivery; intrapartum events; neonatal sex; mode of conception; maternal hypothyroidism; hyperthyroidism; nuchal cord; history of intrauterine fetal demise; preeclampsia; recurrent pregnancy loss; premature rupture of membranes; smoking during pregnancy; consanguineous marriage; oligohydramnios; polyhydramnios; gestational diabetes mellitus (GDM); pregestational diabetes mellitus; preeclampsia in the index pregnancy; gestational hypertension; and chronic hypertension.

Birth weight percentiles were calculated according to gestational age at delivery and neonatal birth weight.

### 2.2. Cardiotocographic Evaluation

Intrapartum fetal heart rate monitoring was performed using either the Philips Avalon FM30 (Philips Medical Systems, Andover, MA, USA) or Sonicaid Team Care Duo (Huntleigh Healthcare Ltd., Diagnostic Products Division, Cardiff, UK) monitoring system with external ultrasound transducers. All cardiotocographic tracings were stored electronically after delivery. CTG recordings obtained during the final hour before delivery were retrospectively reviewed and classified.

Fetal heart rate (FHR) patterns were classified according to the three-tier system proposed by the National Institute of Child Health and Human Development (NICHD) workshop in 2008 [11], which is broadly comparable to the classification subsequently adopted by the International Federation of Gynecology and Obstetrics (FIGO) [12]. Category III tracings were considered indicative of non-reassuring fetal status (NRFS). In addition, persistent Category II tracings associated with recurrent late or variable decelerations, minimal or absent baseline variability, or other features suggestive of evolving fetal compromise were considered indicative of suspected NRFS and prompted operative delivery if they persisted despite appropriate intrauterine resuscitative measures. The decision to proceed with operative delivery was based on the overall clinical assessment, including CTG findings, labor progression, and maternal and fetal condition, and was ultimately made by the attending obstetrician. Accordingly, both NICHD Category II and Category III fetal heart rate tracings were included in the NRFS group, reflecting contemporary clinical practice in which persistent Category II tracings with additional concerning features, as well as Category III tracings, may prompt operative intervention.

Participants were divided into two groups according to the presence of non-reassuring fetal status (NRFS). Cases demonstrating Category II or Category III fetal heart rate tracings during labor were classified as the NRFS group and constituted the study cohort. Patients with Category I tracings throughout labor were included in the control group.

### 2.3. Umbilical Cord Blood Gas Analysis and Neonatal Outcomes

In all cases, umbilical arterial blood pH was measured immediately after delivery. APGAR scores were assessed at 1 and 5 min after birth and, when clinically indicated, at 10 min. These parameters were evaluated as indicators of early neonatal outcomes. Umbilical arterial pH < 7.20 was selected as the primary outcome because it is the most used threshold in studies evaluating the diagnostic performance of cardiotocography [13]. This cutoff represents the lower limit of the normal physiological distribution of umbilical arterial pH (approximately the mean minus two standard deviations) rather than a threshold for severe neonatal injury. Since the primary objective of the present study was to assess the ability of CTG to identify early fetal acid–base disturbance rather than severe metabolic acidemia, pH < 7.20 was considered the most appropriate outcome measure. Moreover, the use of lower pH thresholds (e.g., <7.10) or composite definitions of metabolic acidemia would have resulted in substantially fewer outcome events, thereby limiting the reliability of statistical analyses.

For umbilical cord blood gas analysis, the umbilical cord was doubly clamped immediately after delivery using two Kocher clamps placed at least 10 cm apart. Two milliliters of blood were obtained from the umbilical artery using pre-heparinized blood gas syringes (Genject, Ankara, Türkiye). To prevent air contamination, the syringe was sealed immediately after sampling. Blood gas analyses were performed within 10 min of collection using a self-calibrating automated blood gas analyzer (Siemens Healthcare Diagnostics, Erlangen, Germany).

### 2.4. Inclusion and Exclusion Criteria

Inclusion criteria:

- Maternal age ≥ 18 years.

Exclusion criteria:

- Maternal age < 18 years;
- Multiple pregnancy;
- Neonates with major cardiac or intracranial anomalies.

### 2.5. Statistical Analysis

Statistical analyses were performed using SPSS version 23.0 (IBM Corp., Armonk, NY, USA). Continuous variables were expressed as mean ± standard deviation (SD) or median (minimum–maximum), depending on data distribution. Categorical variables were presented as frequencies and percentages.

Comparisons between the study and control groups were performed using Student’s *t*-test for continuous variables and Pearson’s chi-square test or Fisher’s exact test for categorical variables, as appropriate. Differences in mean umbilical arterial pH across NICHD fetal heart rate categories were evaluated using one-way analysis of variance, followed by Bonferroni post hoc testing for pairwise comparisons.

Multivariable logistic regression analyses using a forward stepwise selection procedure were performed to identify independent predictors of non-reassuring fetal status and umbilical arterial pH < 7.20. Results were reported as odds ratios (ORs) with 95% confidence intervals (CIs). Accordingly, adjusted odds ratios and 95% confidence intervals were only reported for variables retained in the final multivariable model.

The diagnostic performance of cardiotocography for predicting umbilical arterial pH < 7.20 was assessed by calculating sensitivity, specificity, positive predictive value (PPV), negative predictive value (NPV), and overall diagnostic accuracy. A two-sided *p*-value < 0.05 was considered statistically significant.

## 3. Results

Among the 596 women included in the study, 67.1% delivered by cesarean section and 32.9% delivered vaginally. All patients in the study group (non-reassuring fetal status- NRFS) underwent cesarean delivery, whereas 54.8% of patients in the control group delivered by cesarean section and 45.2% by vaginal delivery. The mode of delivery differed significantly between the groups (*p* < 0.05) (Table 1).

Overall, 27.2% of cesarean deliveries were performed because of NRFS. In the control group, the most common indications for cesarean delivery were previous cesarean section (44.0%) and cesarean indications differed significantly between the groups (Table 1). Only seven patients experienced intrapartum complications, all of whom belonged to the control group. Among these cases, 71.4% required operative delivery, whereas 28.6% experienced shoulder dystocia.

In vitro fertilization (IVF) pregnancies accounted for 4.0% of all cases. The proportion of IVF pregnancies was significantly higher in the study group than in the control group (6.8% vs. 3.0%, *p* < 0.05).

Gestational age at delivery ranged from 27 to 41 weeks, with a mean of 37.52 ± 2.41 weeks. The mean gestational age was significantly lower in the study group compared with the control group (36.33 ± 3.66 vs. 37.97 ± 1.51 weeks, *p* < 0.05). In the study group, 17 women delivered between 27 and 30 weeks, 26 delivered between 31 and 34 weeks, and 119 delivered between 35 and 41 weeks. The corresponding numbers in the control group were 2, 5, and 427, respectively. Preeclampsia complicated 3.2% of all pregnancies (Table 2). The prevalence of preeclampsia was significantly higher in the study group than in the control group (8.0% vs. 1.0%, *p* < 0.05). Likewise, gestational hypertension was more common in the study group (3.7% vs. 0.7%, *p* < 0.05).

Birth weight ranged from 490 g to 4570 g, with a mean value of 3059.9 ± 744.2 g. Neonates in the study group had a significantly lower mean birth weight than those in the control group (2572.7 ± 962.7 g vs. 3241.8 ± 542.6 g, *p* < 0.05). The distribution of birth-weight percentiles differed significantly between the groups. The proportions of neonates below the 10th percentile and between the 10th and 50th percentiles were higher in the NRFS group, whereas birth weights above the 50th percentile were more frequent in the control group (*p* < 0.00) (Table 3).

**Figure 1:**
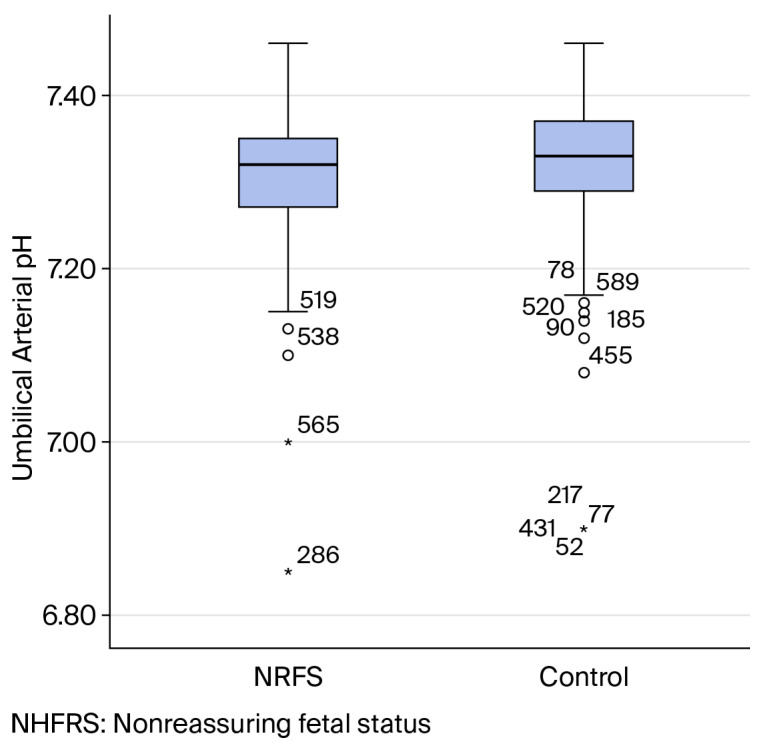
Umbilical Artery pH Values of Groups. *: patients Ph < 7.0.

Umbilical arterial pH values ranged from 6.85 to 7.46, with a mean of 7.32 ± 0.07. Mean umbilical arterial pH was significantly lower in the NRFS group than in the control group (7.30 ± 0.08 vs. 7.32 ± 0.07, *p* < 0.05) (Figure 1, Table 3). Twenty-eight neonates (4.7%) had an umbilical arterial pH below 7.20, of whom 12 (42.9%) belonged to the NRFS group. Mean umbilical arterial pH differed significantly among NICHD fetal heart rate categories (one-way ANOVA, F(2593) = 3.98, *p* = 0.019). The highest mean pH was observed in Category I tracings (7.323 ± 0.072), whereas lower values were observed in Category II (7.301 ± 0.084) and Category III tracings (7.300 ± 0.069). However, pairwise comparisons using Bonferroni correction did not demonstrate statistically significant differences between individual categories.

To identify independent predictors of umbilical arterial pH < 7.20, a multivariable logistic regression analysis was performed using a forward stepwise selection procedure. Variables considered clinically relevant or associated with low cord pH in univariate analyses were entered into the model. The final model was statistically significant (*p* < 0.001), and the retained variables are presented in Table 4. Pregestational diabetes mellitus was independently associated with an 11.71-fold increased risk of umbilical arterial pH < 7.20 (OR: 11.71, 95% CI: 2.63–52.11), whereas preeclampsia increased the risk by 5.14-fold (OR: 5.14, 95% CI: 1.46–18.03).

The 1 min APGAR score ranged from 1 to 10, with an overall mean of 8.34 ± 1.86. The NRFS group had significantly lower 1 min APGAR scores than the control group (6.91 ± 2.27 vs. 8.87 ± 1.34, *p* < 0.05). Similarly, the mean 5 min APGAR score was significantly lower in the study group than in the control group (8.36 ± 1.71 vs. 9.59 ± 0.82, *p* < 0.05) (Table 3). Meconium-stained amniotic fluid was observed in 15.1% of all deliveries. The prevalence was similar between the study and control groups (14.8% vs. 15.2%, respectively; *p* > 0.05).

An additional multivariable logistic regression analysis was performed that included clinically relevant confounding variables (gestational age at delivery, birth weight, oligohydramnios, preeclampsia, and pregestational diabetes mellitus) to further evaluate the robustness of the findings. The results are presented in Supplementary Table S1. Overall, the findings were consistent with those of the primary model. Pregestational diabetes mellitus remained independently associated with umbilical arterial pH < 7.20 after adjustment for the additional covariates, whereas preeclampsia showed a similar direction of association but was no longer statistically significant.

The diagnostic performance of cardiotocography-based identification of NRFS for predicting umbilical arterial pH < 7.20 was also evaluated. Sensitivity, specificity, positive predictive value, and negative predictive value were 42.9%, 73.6%, 7.4%, and 96.3%, respectively. The overall diagnostic accuracy was 72.1%. Among patients who underwent operative delivery for NRFS, 92.6% had an umbilical arterial pH ≥ 7.20, indicating a high false-positive rate (Table 5).

## 4. Discussion

The present study evaluated the relationship between intrapartum cardiotocographic findings, umbilical arterial blood gas parameters, and neonatal outcomes by comparing women with normal CTG tracings and those undergoing operative delivery for suspected non-reassuring fetal status (NRFS). The principal findings were that neonates in the NRFS group had significantly lower umbilical arterial pH values and APGAR scores than controls. In addition, pregestational diabetes mellitus and preeclampsia were identified as independent predictors of umbilical arterial pH < 7.20. Although umbilical arterial pH differed significantly across NICHD fetal heart rate categories, CTG showed limited ability to accurately identify fetal acidemia, with a positive predictive value of only 7.4% despite a high negative predictive value. These findings highlight both the clinical utility and the inherent limitations of intrapartum fetal heart rate monitoring. Overall, our results support the hypothesis that CTG abnormalities are associated with lower umbilical arterial pH values while also demonstrating that CTG alone has limited ability to accurately identify neonates with clinically significant acidemia.

The low positive predictive value of CTG for predicting neonatal acidemia observed in our cohort (7.4%) is consistent with recent evidence demonstrating that no currently available CTG classification system achieves both high sensitivity and high specificity. A recent head-to-head comparison of the FIGO, NICE, and Swedish classification systems demonstrated substantial differences in diagnostic performance, with FIGO-2015 favoring specificity and NICE-2022 favoring sensitivity, underscoring the persistent trade-off between avoiding unnecessary intervention and accurately identifying fetal compromise [14].

These findings are in line with the physiology-based perspective proposed by Pinas and colleagues, who suggested that conventional CTG classification systems rely predominantly on visual pattern recognition rather than direct assessment of fetal pathophysiology [15]. CTG reflects the fetal cardiovascular response to stress rather than tissue oxygenation itself. Therefore, abnormal CTG patterns do not necessarily indicate clinically significant hypoxia or acidemia. Consequently, physiological adaptive responses to intrapartum stress may be misinterpreted as fetal compromise, reducing the specificity of CTG and contributing to its persistently high false-positive rate. They further emphasized that the high false-positive rate of CTG has contributed to increased operative delivery rates without a corresponding reduction in cerebral palsy or perinatal mortality. This highlights the need to interpret CTG findings within the overall clinical context and, whenever possible, in conjunction with objective indicators of fetal condition. Recent advances in machine learning-based CTG interpretation represent ongoing efforts to overcome the limitations of conventional visual assessment [6,7,16]. Although these approaches have shown encouraging results, including improved diagnostic performance and lower false-positive rates in preliminary validation studies, further external validation and prospective clinical evaluation are required before widespread implementation in routine obstetric practice.

In the present study, umbilical arterial pH differed significantly across NICHD fetal heart rate categories. Although lower pH values were observed in Category II and III tracings compared with Category I, the effect size was small and pairwise comparisons were not significant after correction for multiple testing. These findings suggest that worsening fetal heart rate category is associated with lower umbilical arterial pH values; however, NICHD categorization alone may not reliably identify fetuses with clinically significant acidemia. Similarly, although the difference in mean umbilical arterial pH between the NRFS and control groups was statistically significant (7.30 vs. 7.32), its absolute magnitude was small and, in isolation, is unlikely to alter immediate neonatal management. The primary objective of the present study, however, was not to evaluate the clinical impact of small differences in mean pH values but rather to assess the diagnostic performance of CTG in identifying fetuses at risk of acidemia. Consistent with this objective, CTG demonstrated limited diagnostic performance: although all women in the NRFS group underwent operative delivery because of suspected fetal compromise, 92.6% of neonates had an umbilical arterial pH ≥ 7.20, yielding a positive predictive value of only 7.4% despite a negative predictive value of 96.3%. The limited diagnostic performance observed in our study is likely explained, at least in part, by the inherent limitations of Category II fetal heart rate tracings, namely its high false-positive rate, and indicate that CTG findings should be interpreted within the overall clinical context rather than used in isolation when making decisions regarding operative delivery.

One possible explanation for the high rate of normal umbilical arterial pH values observed among patients who underwent cesarean delivery for non-reassuring fetal status in our cohort is the inherent limitation of Category II fetal heart rate (FHR) tracings. Category II tracings encompass a broad spectrum of FHR patterns that are neither clearly reassuring nor definitively pathological and therefore represent the most challenging category to interpret and manage [4,11,17]. Although these tracings warrant close surveillance and clinical reassessment, they are poor predictors of fetal acidemia. Their heterogeneity limits specificity and contributes to substantial interobserver variability. Consequently, management decisions based solely on Category II FHR findings may result in a considerable number of operative deliveries in fetuses without significant acid–base compromise [17]. Previous studies have similarly demonstrated that suspicious and pathological FHR tracings have limited positive predictive value for metabolic acidemia and adverse neonatal outcomes [12,17]. Collectively, these findings reinforce the importance of interpreting CTG within the overall clinical context rather than relying on CTG findings in isolation when making intrapartum management decisions.

Our findings also demonstrated significantly lower 1 min and 5 min APGAR scores in the NRFS group than in the control group, whereas no significant difference was observed in 10 min APGAR scores. Similar findings were reported by Kohli et al., who demonstrated an increased risk of low APGAR scores among neonates delivered due to acute NRFS [18]. Likewise, Singh et al., found significantly lower 1 min and 5 min APGAR scores in the acute fetal distress group compared with controls [19]. Collectively, these findings suggest that fetuses with non-reassuring fetal status are more likely to exhibit transient neonatal compromise at birth, although most neonates show clinical improvement within the first minutes of life.

Although CTG demonstrated limited accuracy for predicting neonatal acidemia in our cohort, more severe CTG abnormalities remain clinically important. Comart et al. similarly reported lower umbilical cord pH values in fetuses with NRFS [20]. Moreover, Category III fetal heart rate tracings have been shown to be significantly more frequent among neonates who subsequently develop hypoxic–ischemic encephalopathy than among controls [21]. Together, these findings suggest that while CTG has limited positive predictive value for neonatal acidemia, severe fetal heart rate abnormalities continue to represent important markers of clinically significant fetal compromise.

Gestational age at delivery was significantly lower in the NRFS group than in the control group. Similar findings have been reported by Wang et al., suggesting an increased risk of intrapartum fetal compromise at earlier gestational ages [22]. This association may be explained by gestational age-related differences in fetal heart rate characteristics and autonomic nervous system maturation, which can influence CTG interpretation [23]. However, other studies have found no significant association between gestational age and fetal distress [20,24]. These discrepancies likely reflect differences in study population, obstetric management, and diagnostic criteria.

Another notable finding was that neonates in the NRFS group had significantly lower birth weights and a higher proportion of birth weights below the 50th percentile, suggesting an association between impaired fetal growth and intrapartum fetal compromise. Previous studies have similarly reported low birth weight as a risk factor for acute fetal distress [19]. In addition, fetal growth restriction and placental dysfunction may modify the clinical significance of CTG abnormalities, as identical fetal heart rate patterns may reflect different degrees of fetal compromise depending on the underlying fetal condition [25]. However, not all studies have confirmed this association, and discrepancies are likely attributable to differences in study population and obstetric management [26]. Furthermore, the lower gestational age at delivery and the higher prevalence of maternal comorbidities, particularly pregestational diabetes mellitus and preeclampsia, may also have influenced neonatal acid–base status. Although multivariable logistic regression was performed to account for potential confounding, residual confounding cannot be excluded. Therefore, CTG findings should be interpreted together with gestational age, fetal growth, and maternal risk factors rather than in isolation.

Multivariable analysis identified independent predictors of umbilical arterial pH < 7.20. The presence of pregestational diabetes mellitus was associated with an 11.71-fold increased risk of umbilical arterial pH < 7.20, whereas preeclampsia increased this risk by 5.14-fold. Similar findings have been reported in the literature. Kapaya et al., in a study including 294 pregnant women, stratified patients according to umbilical arterial pH values (<7.20 vs. ≥7.20) and demonstrated a significantly higher prevalence of pregestational diabetes mellitus among neonates with pH < 7.20 [27]. Likewise, Zeteroglu et al. compared women with mild, severe, and superimposed preeclampsia with normotensive controls and found significantly lower umbilical cord pH values among pregnancies complicated by preeclampsia [28]. These findings support our observation that both pregestational diabetes mellitus and preeclampsia are important risk factors for neonatal acidemia.

The association between pregestational diabetes mellitus, preeclampsia, and an increased risk of fetal acidemia observed in our study may be explained by mechanisms beyond acute intrapartum hypoxia alone. Although contemporary intrapartum fetal monitoring primarily focuses on detecting fetal hypoxia, growing evidence suggests that adverse perinatal outcomes result from the interaction of hypoxia with pathological processes that impair fetal reserve and adaptive capacity [4]. Both pregestational diabetes mellitus and preeclampsia are associated with placental dysfunction, chronic fetal stress, and altered fetal oxygenation, potentially reducing fetal tolerance to intrapartum stress [29]. This concept is supported by studies in pregnancies complicated by preeclampsia, demonstrating an inverse relationship between umbilical cord pH and the burden of fetal heart rate decelerations, with total deceleration burden remaining an independent predictor of acidemia [30]. Consistent with these observations, pregestational diabetes mellitus and preeclampsia emerged as independent predictors of umbilical arterial pH < 7.20 in our multivariable analysis. Taken together, these findings emphasize that assessment of intrapartum fetal well-being should integrate fetal heart rate characteristics with the underlying maternal–placental clinical context to improve identification of fetuses at genuine risk of acidemia.

The relatively high cesarean section rate observed in the control group reflects the referral nature of our tertiary care center. Most cesarean deliveries in the control group were performed for established obstetric indications, including previous cesarean delivery, malpresentation, placenta previa, and cephalopelvic disproportion, rather than suspected fetal compromise. Therefore, the high baseline cesarean rate should not be interpreted as being attributable to CTG findings alone.

Although the mean pH difference was statistically significant, its absolute magnitude was small. This finding should therefore be interpreted together with the overall diagnostic performance of CTG, particularly its low positive predictive value and high false-positive rate, rather than as evidence of clinically important acidemia in every case of NRFS.

## 5. Conclusions

This study has several limitations. First, it was conducted at a single tertiary referral center, which may limit the generalizability of the findings. Second, although the overall cohort included 596 patients, only 28 neonates had an umbilical arterial pH < 7.20, limiting the statistical power for subgroup analyses and multivariable modeling. Although a forward stepwise logistic regression approach was used to reduce the number of variables retained in the final model, the limited number of outcome events may have affected model stability and increased the risk of overfitting. Therefore, the identified independent predictors should be interpreted with caution and require validation in larger prospective studies. Third, because of the limited number of acidemic neonates, more comprehensive multivariable models evaluating the independent contribution of individual CTG features and other obstetric variables could not be robustly developed. Rather than questioning the role of CTG in intrapartum fetal surveillance, our findings emphasize the importance of interpreting CTG within the overall clinical context and reinforce the need for complementary objective measures and improved diagnostic approaches to minimize unnecessary operative intervention. Larger prospective studies incorporating detailed CTG characteristics and contemporary analytical methods are warranted to further clarify the relationship between specific CTG abnormalities and neonatal acidemia.

## Figures and Tables

**Table 1:** Maternal and Obstetrics Demographic Variables of Patients.

	All Patients (N: 596)	NRFS Group (N: 162)	Control Group (N: 434)	*p*
Maternal Age	30.14 ± 5. 62	29.12 ± 6.24	30.50 ± 5.27	0.013
BMI	26.49 ± 3.02	26.59 ± 3.09	26.46 ± 3	0.624
Gravidity	2.43 ± 1.4	1.93 ± 1.29	2.62 ± 1.40	<0.001
Parity	0.96 ± 0.97	0.54 ± 0.91	1.12 ± 0.94	<0.001
IVF Pregnancy	24 (4%)	11 (6.8%)	13 (3%)	0.036
**PPROM**				
Yes	4 (0.7%)	4 (2.5%)	0	0.001
No	592 (99.3%)	158 (97.5%)	434 (100%)	
**Mode of Delivery**				
C/S	400 (67.1%)	162 (100%)	238 (54.8%)	<0.001
Vaginally	196 (32.9%)	0	196 (45.2%)	
**Indication for C/S**				
NRFS	162 (27.2%)	162 (100%)	0	0.001
Previous C/S	191 (32%)	0	191 (44%)	
Malpresentation	25 (4.2%)		25 (5.8%)	
PPT	11 (1.8%)		11 (2.5%)	
Cephalopelvic disproportion	22 (3.7%)		6 (2.5%)	
GA at delivery, weeks	37.52 ± 2.41	36.33 ± 3.66	37.97 ± 1.51	<0.001

Values are given as X ± ss, and number (%).

**Table 2:** Maternal Risk Factors of Patients.

	All Patients (N: 596)	NRFS Group (N: 162)	Control Group (N: 434)	*p*
Smoking	23 (3.9%)	6 (3.7%)	17 (3.9%)	0.904
Oligohydramnios	30 (5%)	19 (11.7%)	11 (2.5%)	<0.001
Polyhydramnios	25 (4.2%)	12 (7.4%)	13 (3%)	0.017
GDM	39 (6.5%)	13 (8%)	26 (6%)	0.372
Pregestational Diabetes	13 (2.2%)	6 (3.7%)	7 (1.6%)	0.120
Chronic HT	23 (3.9%)	13 (8%)	10 (2.3%)	0.001
GHT	9 (1.5%)	6 (3.7%)	3 (0.7%)	0.007
Preeclampsia	19 (3.2%)	13 (8%)	6 (1.4%)	<0.001

Values are given as number (%).

**Table 3:** Neonatal outcomes of all patients.

	All Patients	NRFS Group	Control Group	*p*
Birth Weight *	3059.9 ± 744.2	2572.7 ± 962.7	3241.8 ± 542.6	<0.001
Birth weight percentile **				
<10th prct	113 (19%)	61 (37.7%)	52 (12%)	<0.001
11–50 prst	216 (36.2%)	62 (38.3%)	15 (35.5%)	
50–90 prst	199 (33.4%)	33 (20.4%)	166 (38.2%)	
>90 prst	68 (11.4%)	6 (3.7%)	62 (14.3%)	
Umbilical Arterial pH *	7.32 ± 0.07	7.3 ± 0.08	7.32 ± 0.07	0.007
1 min APGAR *	8.34 ± 1.86	6.91 ± 2.27	8.87 ± 1.34	<0.001
5 min APGAR *	9.25 ± 1.26	8.36 ± 1.71	9.59 ± 0.82	<0.001
10 min APGAR **	6.58 ± 1.26	6.29 ± 1.07	7.4 ± 1.51	0.090
Meconium in amnios **				
Clear	506 (84.9%)	138 (85.2%)	368 (84.8%)	0.905
Stained	90 (15.1%)	24 (14.8%)	66 (15.2%)	
**Nuchal Cord**				
Yes	4 (0.7%)	4 (2.5%)	0	0.001
No	592 (99.3%)	158 (97.5%)	434 (100%)	

Values are given as * number (%) and ** X ± ss, NRFS: Non-reassuring fetal status.

**Table 4:** Multivariate analysis of risk factors for Umbilical Artery pH < 7.2.

	Odds Ratio (95% CI)	*p* Value
Pregestational Diabetes	11.71 (2.63–52.11)	0.001
Preeclampsia	5.14 (1.46–18.03)	0.011

Χ^2^ = 208.91; *p* < 0.001; n = 596; Pseudo R^2^ = 89%.

**Table 5:** Diagnostic Performance of CTG-Based Diagnosis of Non-Reassuring Fetal Status for Predicting Umbilical Arterial pH < 7.20.

Umbilical Arterial pH	NRFS (+) (n = 162)	NRFS (−) (n = 434)
**<7.2**	12(7.4%)	16(3.7%)
**>7.2**	150 (92.6%)	418(96.3%)
Total	162 (100%)	434(100%)
		**Value (95% CI)**
OR		2.09 (95% CI: 0.97–4.52)
Sensitivity		42.9%
Specificity		73.6%
Positive predictive value		7.4%
Negative predictive value		96.3%
Diagnostic accuracy		72.1%

NRFS: Non-reassuring fetal status, OR: odds ratio.

## Data Availability

The datasets used and/or analyzed during the current study are available from the corresponding author upon reasonable request.

## Supplementary Materials

The following supporting information can be downloaded at: https://www.mdpi.com/article/10.3390/jcm15145464/s1, Table S1: Multivariable logistic regression analysis of clinically relevant factors associated with umbilical arterial pH < 7.20.

## Abbreviations

NRFS	Non-Reassuring Fetal Status
CTG	Cardiotocography
NICHD	National Institute of Child Health and Human Development
FIGO	International Federation of Gynecology and Obstetrics
