# Supplementary material for: Association of Intrapartum Cardiotocography Findings with Umbilical Arterial Blood Gas Parameters and Neonatal Outcomes in Non-Reassuring Fetal Status
Source: J Clin Med. 2026 Jul 13;15(14):5464. doi: 10.3390/jcm15145464 (PMC13412630; doi:10.3390/jcm15145464)
Supplement: Supplementary file 1 [file jcm-15-05464-s001.zip › jcm-4412149-supplementary.pdf]

Supplementary Table S1: Multivariable logistic regression analysis of clinically relevant factors associated with umbilical arterial pH <7.20.

**Variable**	**Cases with UA pH <7.20, n (%)***	**Adjusted OR (95% CI)**	**p value**
Pregestational diabetes mellitus	4 (14.2%)	9.07 (2.28–36.08)	0.002
Preeclampsia	4(14.2%)	3.81 (0.88–16.42)	0.073
Oligohydramnios	3 (10.71%)	2.38 (0.61–9.28)	0.210
Gestational age at delivery (weeks)	36.5± 3	0.98 (0.77–1.24)	0.838
Birth weight (g)	2844 ± 807	1.00 (0.999–1.001)	0.872

*For continuous variables, values are presented as mean ± standard deviation.

$\chi^2 = 16.65$, df = 5, $p = 0.005$; Nagelkerke $R^2 = 0.087$; $n = 596$.
